# Spatial–temporal distribution of chikungunya virus in Brazil: a review on the circulating viral genotypes and *Aedes* (*Stegomyia*) *albopictus* as a potential vector

**DOI:** 10.3389/fpubh.2024.1496021

**Published:** 2024-12-11

**Authors:** Maria Eduarda Barreto Resck, Daniel Cardoso Portela Câmara, Flávia Barreto dos Santos, Jefferson Pereira Caldas dos Santos, Barry Wilmer Alto, Nildimar Alves Honório

**Affiliations:** ^1^Laboratório das Interações Vírus-Hospedeiros - LIVH, Instituto Oswaldo Cruz/Fiocruz, Rio de Janeiro, Brazil; ^2^Programa de Computação Científica, Fundação Oswaldo Cruz - PROCC, Fundação Oswaldo Cruz (Fiocruz), Rio de Janeiro, RJ, Brazil; ^3^Instituto de Tecnologia em Fármacos, Fundação Oswaldo Cruz, Rio de Janeiro, Brazil; ^4^Florida Medical Entomology Laboratory-FMEL, University of Florida, Vero Beach, FL, United States; ^5^Núcleo Operacional Sentinela de Mosquitos Vetores-Nosmove/Fiocruz, Rio de Janeiro, Brazil

**Keywords:** chikungunya, virus diversity and genotypes, *Aedes albopictus*, vector competence, environmental/ecological determinants

## Abstract

Chikungunya virus (CHIKV) is mainly transmitted by the invasive mosquito *Aedes* (*Stegomyia*) *aegypti* in tropical and subtropical regions worldwide. However, genetic adaptations of the virus to the peri domestic mosquito vector *Aedes* (*Stegomyia*) *albopictus* has resulted in enhanced vector competence and associated epidemics and may contribute to further geographic expansion of CHIKV. However, evidence-based data on the relative role of *Ae. albopictus* in CHIKV transmission dynamics are scarce, especially in regions where *Ae. aegypti* is the main vector, such as in Brazil. Here, we review the CHIKV genotypes circulating in Brazil, spatial and temporal distribution of Chikungunya cases in Brazil, and susceptibility to infection and transmission (i.e., vector competence) of *Ae. albopictus* for CHIKV to better understand its relative contribution to the virus transmission dynamics.

## Introduction

The dramatic emergence and spread of arboviral diseases in the past 50 years has highlighted the urgent need to review surveillance and control strategies. Only by application of integrated management approaches, emergent epidemic arboviral diseases, such as those recently observed after the emergence of Zika virus (ZIKV) in the Americas, Yellow Fever (YFV) in Angola and Brazil, West Nile virus (WNV) in the Americas and chikungunya virus (CHIKV) worldwide will be able to be prevented and controlled (1, 2).

In the sylvatic cycle, CHIKV usually circulates between non-human primates, other mammalian reservoir hosts and *Aedes* mosquitoes, but in the urban cycle, the virus is transmitted to humans through infectious bites by invasive mosquitoes, mainly *Aedes* (*Stegomyia*) *aegypti* (Linnaeus 1762) (3, 4).

Chikungunya fever is mainly characterized by fever, rash, and incapacitating arthralgia, with symptoms apparent in approximately 80% of the patients (5–7). High attack rates have been observed during outbreaks and more than 30% of the infected individuals develop chronic disease (e.g., debilitating arthralgia and arthritis) that can persist for years (8, 9). Chronic arthralgia often causes significant disability, hindering daily activities and leading to physical and mental distress. Patients frequently report symptoms like appetite loss, poor sleep, mood swings, and depression (10, 11). These symptoms can result in missed work or school, potentially leading to job loss or academic withdrawal, and a reduced quality of life (11–13).

Animal model studies suggest that chronic CHIKV disease may be the result of induced autoimmunity or viral persistence in joint-associated tissues (14). In rare instances, CHIKV infections have also been associated with neurological manifestations (15, 16).

## A brief history of chikungunya

Chikungunya virus (CHIKV), an arbovirus transmitted by mosquito vectors, was first isolated from a febrile patient during an outbreak on the Makonde plateau in southern Tanzania in 1952 and, in 1953, the virus was first isolated from *Ae. aegypti* mosquitoes. Based on the disabling and debilitating symptoms presented, the disease was named chikungunya, derived from the Kimakonde language, and meaning “that which bends up” (17–19).

In 1958 and following years, cases of chikungunya were reported in Uganda and in other sub-Saharan African countries. The existence of a wild cycle was indicated by the isolation of CHIKV from a pool of the forest mosquito *Ae. africanus* collected in the Zika Forest and subsequent infection studies using mice and rhesus monkeys (20). The presence of anti-CHIKV antibodies in experimentally infected vervet monkeys (*Chlorocebus pygerythrus*) was evidence for the possible role of non-human primates and consideration of the involvement of non-human primates in a sylvatic transmission cycle (21).

Since its discovery in Tanzania in the 1950s, CHIKV has been responsible for emerging and reemerging epidemics in several temperate and tropical regions of the world, particularly in geographical areas inhabited by *Ae. aegypti* and *Ae. albopictus* mosquitoes (Skuse 1894) (22–24). The first CHIKV record outside Africa occurred in Thailand, associated with *Ae. aegypti*, in 1958, and India and Cambodia (25). Between the 1970s and 1990s, enzootic outbreaks occurred in and around Senegal (26, 27). Subsequently, CHIKV has emerged and spread across five continents at an unprecedented rate causing millions of cases, mainly in the tropical and subtropical regions worldwide (28, 29). In 2004 an intense epidemic reached the islands of the Indian Ocean. On La Reunión Island more than 300,000 cases were reported in 2006 (30, 31). During the outbreak on islands in the Indian Ocean and Asia, there were reported imported cases of chikungunya in Europe and the Americas (32, 33).

Due to the global epidemiological situation, the Pan American Health Organization (PAHO) began containment planning for a possible introduction of CHIKV in the Americas since 2010 (34, 35) and in that year, CHIKV imported cases were reported in Brazil. However, local transmission of CHIKV in the Americas was first reported in the Caribbean in 2013, on the island of Saint Martin (36). In the following year, nine islands in the Caribbean already recorded more than 15,000 suspected cases and by August 2014, there were more than 1,000 suspected cases of the chikungunya fever in Colombia (37). In April 2015, more than 1 million suspected cases and 191 deaths had already been reported in the Americas (2) and autochthonous transmission had been confirmed in more than 50 territories in the region (37, 38).

## Chikungunya virus genotypes and its relation to emergence and spread

CHIKV belongs to the *Togaviridae* family, genus *Alphavirus* and is a small spherical enveloped virus (60–70 nm diameter) with a genome comprised of a positive single-strand RNA of approximately 11.8 Kb. The genome consists of two open-reading frames (ORFs) that encodes four conserved nonstructural proteins (nsP 1–4), a capsid protein (C), two envelope glycoproteins (E1 and E2) and two cleavage products (E3 and 6 K) (39, 40). NsP1, nsP2, and nsP4 are involved in RNA capping, helicase/protease activity and polymerase activity, respectively (41). NsP3 plays a role in viral replication (42). E1 and E2 proteins are present at high levels in humans during the acute phase of the disease (43, 44). Both 5′ and 3′ untranslated regions are present in the genome with the latter exhibiting stem-loop structures and repeats probably associated with virus adaptation to mosquitoes (45).

Chikungunya virus has a single serotype with four distinct genotypes: West African, East-Central-South-African (ECSA), Asian, and Indian Ocean Lineage (IOL). The geographic expansion of the different CHIKV genotypes was facilitated by the intense circulation of viremic travelers between countries, including temperate regions in Europe and the United States (46). Evolutionary studies provided evidence that the West African genotype originated in Africa, and subsequently spread into Asia, where it evolved into a distinct variant - the Asian genotype. The CHIKV strains from the Reunion Island epidemic in 2005, evolved and were characterized as the distinct ECSA genotype. The ECSA strains reemerged from the mainland in East Africa during an outbreak in Kenya and spread to Indian Ocean islands and India (47). This led the virus to reach La Reunion island, when its geographic range expanded rapidly to include several countries in Europe (32, 48), Americas (49) and Asia (50). The new Indian Ocean Lineage (IOL) evolved independently, as an ECSA monophyletic group (26, 27, 46, 51, 52). Both Asian and ECSA genotypes are those most frequently detected following the virus spread worldwide (53).

The CHIKV ECSA genotype identified during the Indian Ocean epidemic was a mutant, with a substitution from alanine to valine at position 226 of the E1 envelope glycoprotein (E1-A226V) and was described as the IOL (54, 55). The E1-A226V mutation enhanced infectivity (i.e., lower oral infection dose of 50% of mosquitoes tested, OID_50_), viral dissemination efficiency to secondary organs, and transmission by *Ae. albopictus* to mice, favoring it as the main vector in the region (31, 55, 56). The high density of *Ae. albopictus* in Réunion Island and lower viremia necessary to infect the local *Ae. albopictus* population (i.e., lower viremic thresholds occur sooner and are sustained for longer periods in human hosts) contributed to viral spread in this region. During this time, 255,000 cases of CHIKV were reported from March 2005 to April 2006, with the IOL strain identified in 90% of the isolates from human cases (54, 57, 58). The extensive geographic distribution of *Ae. albopictus,* coupled with mutations that improve fitness and infectivity of CHIKV in *Ae. albopictus*, may allow for expansion of CHIKV into temperate ecosystems (59), as observed with small outbreaks in France and Italy (32).

Tsetsarkin et al. (55) demonstrated that the E1-A226V mutation results in a ECSA genotype being 100-fold more infectious for *Ae. albopictus*. However, this mutation is associated with a reduction in infectivity of CHIKV in *Ae. aegypti* midguts, which is considered the main vector of CHIKV, but not in Réunion Island. This CHIKV strain has spread across Asia, although there are also circulating CHIKVs in the region that do not carry the mutation (60).

A new classification for the ECSA genotype was proposed by Schneider et al. (61), given its wide expansion and identification of distinct strains/genotypes. According to the authors, ECSA genotype could be divided into 3 different genotypes, one for each region of the African continent indicated in its name (61).

## Spatial–temporal distribution of chikungunya in Brazil

In Brazil, CHIKV autochthonous transmission occurred after the simultaneous introduction of the Asian and ECSA genotypes in 2014 in the municipality of Oiapoque, Amapá, North region and Feira de Santana, Bahia, Northeast region, respectively (37, 52). In the following years, the ECSA genotype spread to other Brazilian states, and CHIKV outbreaks were registered in the Northeast [Bahia (62–64); Alagoas (65, 66), Piauí (67) Sergipe (68, 69), Maranhão (70)], North [Roraima (71)], Southeast [Rio de Janeiro (72–75) and Minas Gerais, (76)], and Midwest regions (77). Despite reports on the south region, most cases in 2014 and 2015 were imported from the other Brazilian regions (78). Since 2016, Brazil has been the epicenter of CHIKV epidemics in the Americas with reports of annual outbreaks each year and more than 1.6 million cases to date (79).

A previous study reported that the ECSA genotype was likely introduced to Rio de Janeiro early in 2014 through a single event, after primary circulation in Bahia in the previous year (80). On the other hand, Fabri et al. (75) suggests that two independent introductions of the ECSA genotype occurred in Rio de Janeiro between 2016 and 2019, both from the Northeast region (75), showing the complexity of tracing an entry path of CHIKV in Brazil. In fact, a recent study analyzing the epidemiological patterns of the designated ECSA American sub-lineage in Rio de Janeiro revealed two distinct clades introduced from the Northeast region mid-2015 and mid-2017 (81). Despite the detection of both Asian and ECSA genotypes in Brazil, the latter was more frequently associated with symptomatic cases in the country (71, 82, 83). Moreover, the newly emergent ECSA American sub-lineage has become prevalent throughout Brazil (79). Previous studies on lineage replacement among dengue viruses (DENV) supports the notion that differences in underlying viral fitness, as measured by viraemia levels in humans and relative infectivity in mosquitoes, is the main driver of evolutionary events where lineage turnover has occurred, most notably with Southeast Asian genotypes of dengue virus displacing American genotypes (84–89).

Epidemics caused by CHIKV present a cyclical pattern, which can be characterized by periods of epidemiological silence, rotated with periods of intense viral circulation. Both Asian and ECSA genotypes could spread and co-circulate in Brazil, considering suitability of *Ae. aegypti* and *Ae. albopictus* vectors. The rapid spread and establishment of CHIKV was facilitated by the high density of its main vector *Ae. aegypti*, favorable climate, unplanned urbanization, wide availability of reservoirs, human behavior, and a large population of susceptible human hosts (22, 54, 57, 58, 90–96).

Additionally, the simultaneous circulation of CHIKV, DENV, Zika virus (ZIKV), and other arboviruses of medical importance [e.g., Mayaro (MAYV) and Oropouche viruses (OROV)], represents a serious public health challenge for Brazil, because of overlapping clinical signs and symptoms, unavailability of specific and reliable tests for differential diagnosis for health professionals, as well as highlighting the need for active and efficient arbovirus surveillance (37, 97–99).

## Temporal analysis of CHIKV

**Figure 1:**
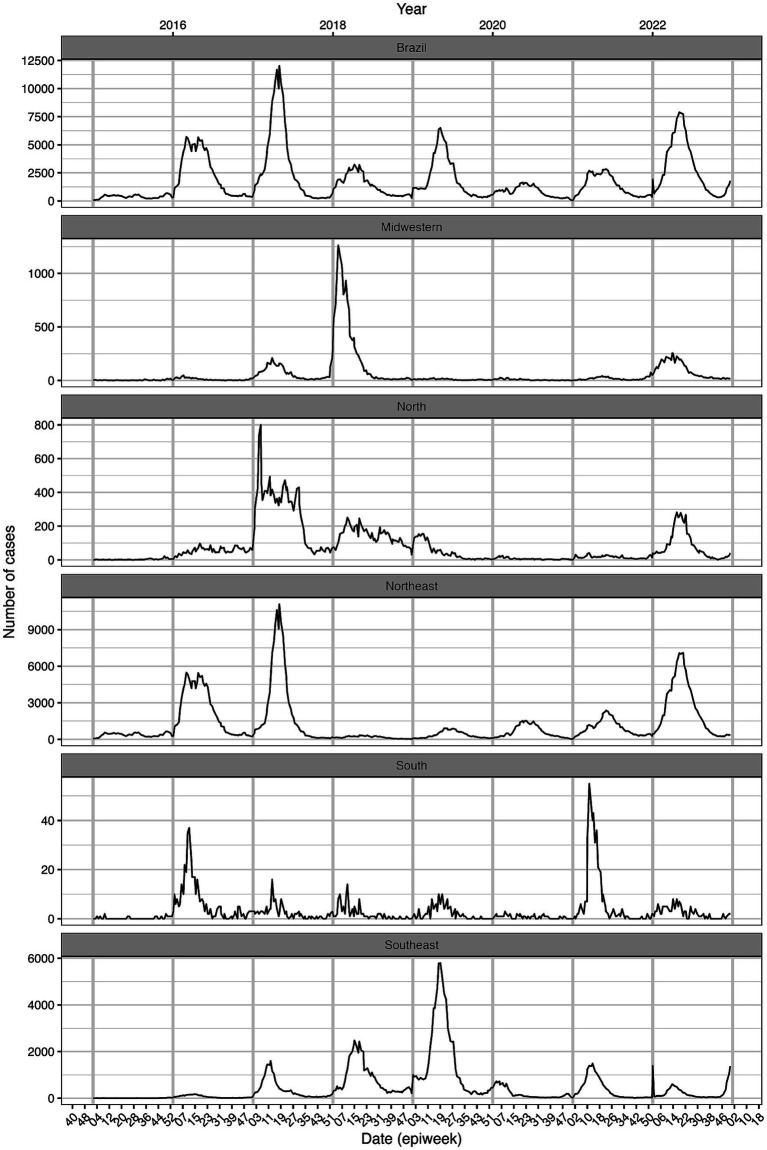
Time series of probable chikungunya cases in Brazil and in each of the five regions of the country: Midwest, Northeast, North, Southeast and South. Notice that the y-axis is not standardized. Source: The Brazilian national disease notification system (SINAN).

Figure 1 shows the time series of probable chikungunya cases in Brazil (top grid) and in each of the country’s five regions (Midwest, Northeast, North, Southeast and South). The y-axes are set at different ranges (i.e., not standardized) which facilitates observing peaks of cases in each region. Despite several peaks of probable cases in the country occurring in the first half of 2016, 2017, 2019, and 2022, it is possible to see that each region sustained different temporal epidemiological patterns. The Northeast region was the most affected of all five regions both in total number of cases (502,761) but also by being the first to display an increase in cases in 2016 followed by a peak of cases in 2017 (with 11,087 cases in epiweek 18–2017). The increase of cases in 2016 in the Northeast region was followed by a peak of cases in the same region during 2017. Similarly, a peak of cases was observed in the North region (801 cases in epiweek 5–2017, out of a total of 32,485 cases in the whole period) and the first of three consecutive growing peaks of cases in the Southeast region (the most densely populated region in Brazil). The Midwest region exhibited the peak of cases in 2018 (1,263 in epiweek 4–2018, out of 24,038 during the whole period), and a new peak of cases in the Southeast region. The year 2019 was the last year exhibiting an increase of cases, with a peak observed in the Southeast region after two consecutive years of growing number of cases (peak of 5,794 in epiweek 19–2019, out of 191,670 cases during the whole period). After 2 years of relatively low number of cases, 2022 saw an increase of cases in the Midwest, Northeast, and North regions of the country. Finally, it is possible to notice the quick increment of cases by the end of 2022 in the Southeast region. The South region (1,285 total number of cases), historically with low numbers of urban arbovirus cases in Brazil, exhibits a noisy pattern of cases, being the only region to exhibit a peak in 2021, but also a substantial number of cases in 2016. With the observed pattern of peaks in each region differing between the years, it is possible to infer that different epidemiological and/or ecological determinants might be regulating such patterns. Since Brazil is a continental country encompassing several different latitudes and biomes, climate and vector ecology might be impacting the transmission patterns. Further studies are needed to provide insight into the heterogenous patterns of CHIKV epidemics between geographic regions. Vector competence, human population immunity, case detection, virus introduction and dispersal, among others, might help elucidate such patterns.

## Spatial analysis of CHIKV

**Figure 2:**
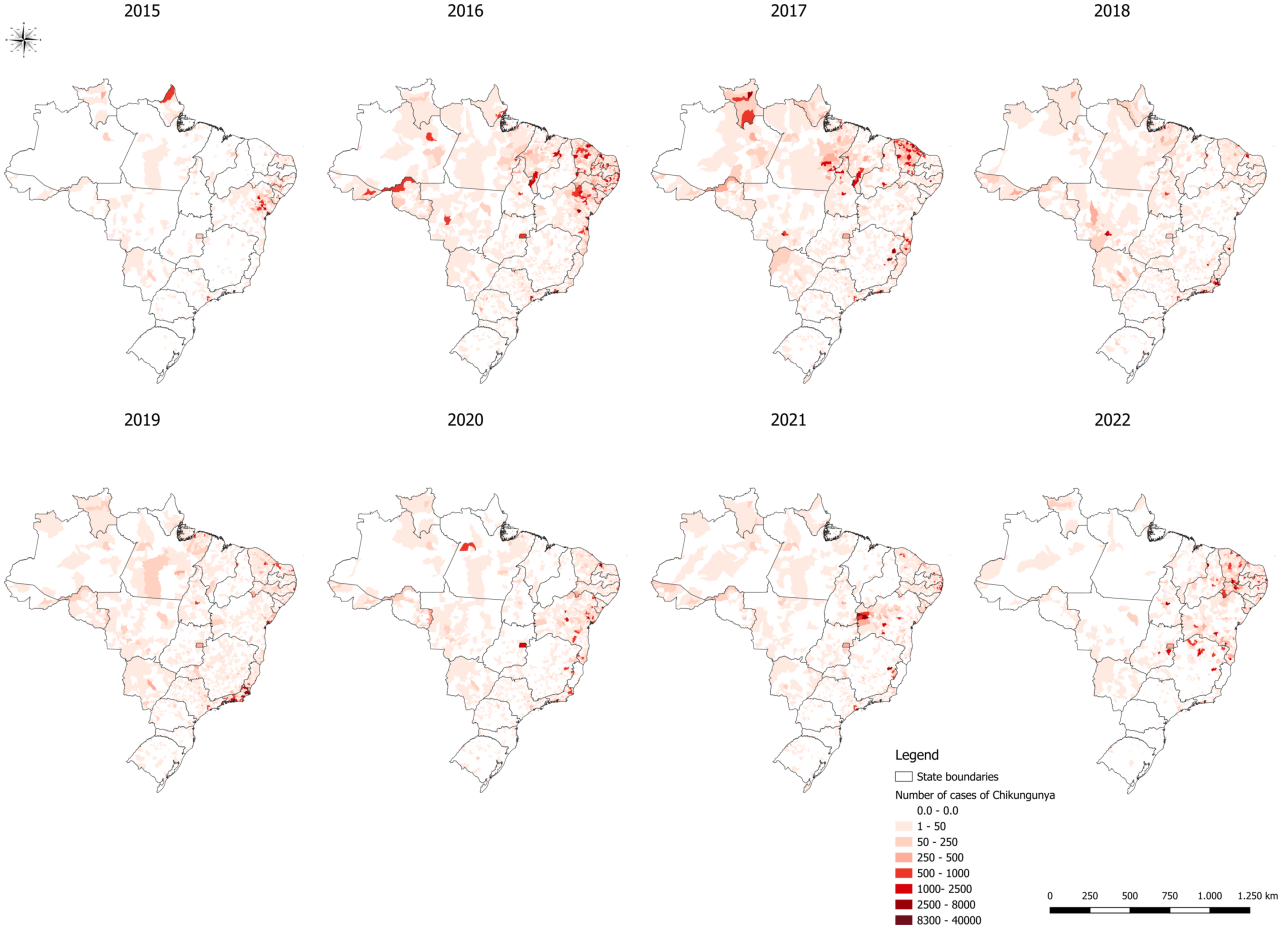
Spatial distribution of probable chikungunya cases in Brazil, 2015–2022. Source: The Brazilian national disease notification system (SINAN).

Figure 2 shows the spatial analysis of probable chikungunya cases in Brazil. The epidemiological elements of chikungunya fever can be discerned from the analysis of disease dissemination patterns over time. The initial occurrence of chikungunya in 2015 in Bahia and Amapá states represents a relevant epidemiological milestone. In the subsequent years, a notable concentration was observed in states within the Northeast region, specifically in 2016, with a focus on Bahia and Ceará, and in 2017, when the concentration shifted primarily to Ceará, along with the emergence of an additional focus in the Northern region, encompassing Tocantins, Southeast Pará, and Roraima (Figure 2).

In 2018 and 2019 the distribution of the phenomenon predominantly centered around the state of Rio de Janeiro, with a notable decrease in its occurrence in other regions of the country. In 2020 and 2021 a resurgence of the phenomenon was observed, but with a distinct geographical distribution. It concentrated in the central and western portions of Bahia, in contrast to the patterns observed in 2016 and 2017. In 2022, the last year analyzed, a new resurgence of the phenomenon was noted in the state of Ceará and in the hinterlands of Pernambuco, as well as an expansion toward the state of Minas Gerais, specifically in its Northern region.

The spatial and temporal analysis revealed complex patterns in the dissemination of the studied phenomenon. Its initial occurrence in Bahia and Amapá in 2015 was followed by periods of concentration in different states, with significant changes in geographical distribution over the years. These variations may be related to a range of factors, including climatic, socioeconomic, and environmental factors.

## Aedes albopictus

Like *Ae. aegypti*, *Ae. albopictus* is an important vector for arbovirus transmission to humans and acts as a primary vector of dengue virus in several countries, where the main vector (*Ae. aegypti*) is absent or rare (100–102). *Aedes albopictus* exhibits eclectic feeding behavior, taking blood meals from diverse animal taxa. Both *Ae. albopictus* and *Ae. aegypti* use artificial container habitats for breeding and lay eggs that can withstand dry conditions, allowing them to survive during unfavorable times of the season and facilitating geographic expansion (103–106). Evidence from field surveys (107–109) and experimental infection studies (110, 111), demonstrate vertical transmission of CHIKV from *Ae. albopictus* adult females to offspring, allowing for the possibility of viral maintenance during adverse environmental conditions such as drought. Also, gonotrophic discordance exhibited by *Ae. albopictus* (and *Ae. aegypti*) is predicted to strongly influence vectorial capacity (112, 113). In fact, *Ae. albopictus* has been shown to be an important and competent vector of more than 20 arboviruses, such as CHIKV, Mayaro, Japanese encephalitis, Rift Valley, West Nile and Sindbis viruses, having the ability to become infected with those viruses, although *Ae. albopictus* is not considered primary vectors of these arboviruses (101, 114–118). Table 1 highlights key biological traits of *Ae. albopictus* that may contribute to the role of this mosquito species as a potential vector of CHIKV.

**Table 1:** Key biological traits of *Aedes albopictus*.

*Aedes albopictus* biological traits	Studies
Feeding behavior	(131, 186–190)
Competitive advantage (larvae)	(191–195)
Vector competence	(115, 146, 152, 196–198)
Dispersal and dispersion	(199–203)
Satyrization (adults)	(204–206)
Vertical transmission	(111, 121, 207–210)

Known as the Asian tiger mosquito, *Ae. albopictus* was first described in Calcutta, India, and is native to Southeast Asia and islands of the Western Pacific and Indian Ocean (119). *Aedes albopictus* is an exotic species that spread throughout the tropics from its native home range by human trade developments (e.g., international tire trade) (120). *Aedes albopictus* is one of the most commonly recognized black-and-white mosquitos, characterized by white bands on its legs, a median longitudinal stripe of silvery scales on the mesonotum and unscaled clypeus (121, 122). It is considered one of the most invasive species worldwide (119, 123, 124). *Aedes albopictus* can be more commonly found in areas with higher vegetation coverage and more scattered human populations, but it was also described in transitional environments with relatively low vegetation cover and frequently coexisting with *Ae. aegypti* (123, 125–128). It has strong plasticity, adapting to several habitats including human environments like urban and suburban areas. Despite being an opportunistic and zoophilic mosquito, when given the opportunity and choice, *Ae. albopictus* shows a predilection for feeding on humans over other animals (107, 129). The predilection of *Ae. albopictus* to feed on humans elevates its potential role as a CHIKV vector, especially in urban areas with high numbers of people and where *Ae. aegypti* is absent.

Blood meal analysis of *Ae. albopictus* at 10 distinct urban-forest interfaces in Brazil showed most blood meals were derived from mammalian hosts, with humans being disproportionately fed upon (130). Similarly, a feeding index measurement showed that *Ae. albopictus* commonly fed on humans and cattle in a 2-year survey in Tremembé County, State of São Paulo, Brazil (131). Patterns of host use where a majority of bloodmeals are derived from humans suggest *Ae. albopictus* has the potential to play a substantial role in CHIKV epidemiology. This anthropophilic trait (i.e., feeding on humans) enhances its invasiveness into human-dominated environments and is an important parameter in vectorial capacity and in determining risk of transmission for arboviruses to humans (132). Moreover, *Ae. albopictus* exhibits gonotrophic discordance, whereby a mosquito engages in multiple feedings during a single gonotrophic cycle, which acts to increase vectorial capacity. Beside feeding on humans, *Ae. albopictus* has a wide variety of hosts, including other mammalians, birds and reptiles which is considered a serious public health threat as it may be a bridge vector for many zoonotic pathogens to humans in Brazil (130, 133, 134). Moreover, *Ae*. *albopictus* mosquitoes have successfully established populations in temperate climates (101, 135), and climate change may further impact its geographic range (103, 136).

*Aedes albopictus* was introduced in Brazil in 1986 in the states of Rio de Janeiro, Minas Gerais, and São Paulo, located in the Southeast region of country (122, 137, 138). Subsequently, this invasive mosquito rapidly spread and expanded to other regions of the country. In 1996, *Ae. albopictus* was recorded for the first time in the Southern and Northern regions of Brazil in Paraná and Amazonas state, respectively (139, 140). One year later, the mosquito was identified in the state of Mato Grosso do Sul in the Midwest region of Brazil (141).

In the Northeast region of Brazil, *Ae. albopictus* was confirmed in Pernambuco in 1999 (142). By this time, it was estimated *Ae. albopictus* was already present in 14 Brazilian states (143). Before 2002, only seven states did not describe the presence of this mosquito species: Acre, Amapá, Roraima, Tocantins, Piauí, Ceará and Sergipe (144), which 10 years later changed this status to three states with no record of occurrence (100). Currently, *Ae. albopictus* can be found in all the 27 Brazilian states (121, 145).

## Vectorial capacity and vector competence

Aiming to measure the rate of disease transmission by bloodsucking insects, previous studies proposed a mathematical model originally created for malaria vectors but later applied to other diseases, that would formulate epidemiological predictions and assess the impact of vector control strategies (146–149). Vectorial capacity, established by Garret-Jones in 1964 (150), is defined by the number of infections that a population of a given vector may distribute per day and considers several entomological parameters, such as, vector abundance and mortality, blood feeding behavior, extrinsic incubation period of the pathogen in the vector, and vector competence of the vector for the pathogen.

Vector competence is the innate capacity of a vector to acquire a pathogen, replication of the pathogen, and transmission after exposure (151). Vectorial capacity is an index controlled by genetic characteristics of the vector and pathogen and environmental conditions (152–154). For efficient transmission to occur, several factors such as arthropod and vertebrate hosts, arbovirus, and environmental conditions must converge (155).

*Aedes aegypti* and *Ae. albopictus* competencies to CHIKV may vary depending on their geographical origin and viral genotype involved in the infection. Regardless, some reports point out *Ae. albopictus* as more competent than *Ae. aegypti* (146, 152, 156–161). The demographic history of *Ae. albopictus* populations is the result of historical lineage diversification and divergence that associates with this species vector competence for CHIKV (162). The vector competence of *Ae. albopictus* populations are a key parameter in assessing the transmission and spread of a disease like chikungunya. Although the ECSA genotype of Brazilian CHIKV does not have the mutation related to adaptability to *Ae. albopictus* (163), which will be further addressed below, other mutations may be present and deserve further investigation.

The spread of the ECSA genotype in Brazil, which apparently replaced the Asian genotype in the Roraima state (Amazon region), suggests a greater potential for transmission of the ECSA genotype (71, 72, 164). In contrast, the CHIKV-Asian genotype has been geographically restricted mostly in the state of Amapá, in the North region (165). According to de Oliveira Ribeiro et al. (165), the pattern of CHIKV strain replacements in the Amazon region could be probably affected by different factors, including the ecological community, human behavior, and the genetics of the virus. An analysis of salivary glands and saliva from *Ae. albopictus* and *Ae. aegypti* infected with CHIKV strains R99659 (Asian genotype) and LR2006 OPY1 (IOL from ECSA genotype) (166) showed that, despite similar and high midgut infection and disseminated infection of these genotypes in both species, transmission efficiency was consistently lower for the Asian genotype in multiple strains of *Ae. albopictus* and *Ae. aegypti*, suggesting a salivary gland exit/escape barrier to the Asian genotype (166). However, both the Asian genotype and the ECSA genotype can spread and co-circulate in the country, considering the suitability of the vector species which includes widespread distribution of both *Ae. aegypti* and *Ae. albopictus* (52, 167).

Regarding *Ae. albopictus* vector competence for CHIKV, Vega-Rúa et al. (115) tested American and Brazilian populations of *Ae. aegypti* and *Ae. albopictus* mosquitoes for their susceptibility to three CHIKV genotypes. All populations were found to be susceptible to infection. However, CHIKV transmission efficiency varied significantly across populations, ranging from 11.1 to 96.7%. Some populations of *Ae. albopictus* from Rio de Janeiro for example were particularly efficient, transmitting infectious viral particles as early as 2 days post-infection. These observations suggest that efficacy of the salivary gland barrier(s) is heterogeneous for *Ae. albopictus* and associates with geographic origin.

In a 2018 study by Honório et al. (152), Brazilian *Ae. albopictus* populations were assessed for their viral dissemination rates after exposure to the CHIKV Asian genotype (GenBank accession: KJ451624) at 2-, 5-, and 13-days post-infection. The results indicated that a significant proportion (exceeding 80%) of individuals from both *Aedes* species developed a disseminated infection as early as 2 days post-exposure. For *Ae. albopictus*, Brazilian populations from Manguinhos, Rio de Janeiro demonstrated a disseminated infection rate of 82.7 ± 7.1% on the second day. Transmission rates were observed to vary across populations, with Brazilian *Ae. albopictus* exhibiting the highest percentage of individuals (82%) capable of transmitting CHIKV by bite (i.e., infected saliva) within 2 days post-infection.

From 2015 to 2020, the range of *Ae. albopictus* in Brazil expanded significantly. In 2020, it was present in 24.8% of the 2,937 surveyed municipalities (728 municipalities). Currently, this mosquito species has been detected in all Brazilian states (145) and natural infection by other arboviruses of medical and veterinary importance has been reported, such as DENV and ZIKV during an outbreak in a rural area in Brazil (168, 169). While its spread seems to be stabilizing, ongoing monitoring programs are crucial to accurately assess its distribution. In fact, authorities should implement control strategies also for *Ae. albopictus*, with regular surveys conducted in all Brazilian municipalities (170).

## Chikungunya virus mutations impacting vector competence

Although CHIKV is generally transmitted by *Ae. aegypti* mosquitoes, the outbreak occurred in La Réunion island was caused by *Ae. albopictus,* which acted as the main vector (171, 172) due to the ECSA CHIKV genotype adaptation to this vector, as the E1-A226V, resulted in a dramatic increase in infectivity.

As a result of the increased viral fitness and vector competence, the virus transmission spread to temperate areas and caused epidemics in regions that lack the typical vector, *Ae. aegypti* (55, 173). In fact, the emergence of the E1-A226V mutated CHIKV during the La Reunion outbreak has been considered a well-characterized example on how a single nucleotide mutation in a virus may lead to a new epidemiological scenario given specific ecological conditions (174). Furthermore, the E1-A226V was associated with enhanced viral dissemination and higher viral loads in *Ae. albopictus* (56), but did not affect viral replication in *Ae. aegypti* (55, 173). However, when the mutation G60D in the E2 gene is in the presence of either alanine or valine at position 226 in E1 in the ECSA genotype, an increased CHIKV infectivity is observed in both *Ae. albopictus* and *Ae. aegypti.* The mutation I211T on E2 also increases viral infectivity in *Ae. albopictus*, but only when associated with E1-A226V (175). Interestingly, despite the long history of circulation in native areas of *Ae. albopictus*, CHIKV Asian genotypes have not shown the E1-A226V mutation yet (27). Lineage-specific epistatic interactions between substitutions of amino acids in positions 226 and 98 of the E1 envelope glycoprotein have largely contributed to an adaptive constraint of the Asian genotype in *Ae. albopictus* (176), underscoring the importance of how different adaptive landscapes can profoundly influence evolution and the emergence of closely related viral genotypes.

It has been shown that the E1-A226V mutation is impacted by the presence of both E1-98T and E2-211I residues found in most ECSA strains, including those circulating introduced in Brazil, thus impacting the potential emergence of CHIKV via *Ae. albopictus* transmission in parts of Africa and the Americas (175). However, it is not yet clear if ECSA strains circulating in Brazil and introduced from Angola in 2014, will select for E2-I211T, as did early IOL strains in Kenya in 2004 (47) and therefore, enabling subsequent adaptive evolution to *Ae. albopictus* (177), posing a major public health challenge.

The E1-A226V evolutionary adaptation illustrates a favorable viral strategy to improve its transmissibility by the vector (174), but other viral mutations are believed to be part of that strategy. It has been shown that mutations on the CHIKV envelope genes from the IOL strains, such as K252Q, K233E, L210Q in the E3 gene and R198Q/S18F in the E3/E3 may impact the initial infection of the *Ae. albopictus* midgut cells (12, 29, 163, 173), and by extension, viral dissemination to secondary organs and transmission. In fact, the substitutions L210Q and K252Q on E2, are associated with a higher CHIKV dissemination in *Ae. albopictus* (163, 173), however, the substitution K211E in the E1 gene, and V264A, in E2 resulted in an increased CHIKV dissemination and transmission in *Ae. aegypti,* but not in *Ae. albopictus* (178).

The 3’UTR plasticity of alphaviruses may also be associated to enhanced transmission and epidemic potential of viral strains. Although it has been reported that the 3’UTR enhances viral replication in a mosquito-specific manner *in vitro* (179), information on the role of this region on virus transmission by mosquito vectors are still scarce. A study on the 3’UTR from strains of the CHIKV Asian genotype showed that mutations occurring at that region could also contribute to vector adaptability (180). For example, infection studies with the Asian genotype of CHIKV in *Ae. aegypti* showed that duplication of repeated RNA elements in the 3’UTR contribute to replication kinetics and subsequent disseminated infection and transmission (181). The authors of this study suggested that mutant viruses with slower replication were less able to cause systemic infections.

Finally, the CHIKV adaptation to *Ae. albopictus* has been consistently associated with the spread of the disease to new areas of the world (177, 182–184), but to date, CHIKV ECSA genotypes circulating in Brazil and leading to adaptive changes in *Ae. albopictus*, such as E1-A226V and E2-L210Q have not been reported yet (12, 72, 164). On the other hand, CHIKV ECSA genotypes circulating in the country presented exclusive amino acids substitutions—V156A and K211T (72), and using a mosquito-to-mouse transmission model, it has been suggested that those mutations may play an important role in CHIKV biology, such as modulation of virus attachment and fusion (185). As RNA viruses present high mutation rates leading to genetically and potentially emergent viral genotypes, and the interaction with the host as well distinct ecological conditions may shape how those will evolve and impact its transmission, studies of molecular characterization of viral strains and vector biology are still needed.
